# Structural Basis of Inhibition of Human Insulin-Regulated Aminopeptidase (IRAP) by Benzopyran-Based Inhibitors

**DOI:** 10.3389/fmolb.2021.625274

**Published:** 2021-04-01

**Authors:** Sudarsana Reddy Vanga, Johan Åqvist, Anders Hallberg, Hugo Gutiérrez-de-Terán

**Affiliations:** ^1^Department of Cell and Molecular Biology, BMC, Uppsala University, Uppsala, Sweden; ^2^Department of Pharmaceutical Chemistry, BMC, Uppsala University, Uppsala, Sweden; ^3^Science for Life Laboratory, Uppsala University, Uppsala, Sweden

**Keywords:** free energy perturbation (FEP), linear interaction energy (LIE), molecular dynamics (MD), Insulin regulated aminopeptidase (IRAP), benzopyran

## Abstract

Inhibition of the insulin-regulated aminopeptidase (IRAP) improves memory and cognition in animal models. The enzyme has recently been crystallized and several series of inhibitors reported. We herein focused on one series of benzopyran-based inhibitors of IRAP known as the HFI series, with unresolved binding mode to IRAP, and developed a robust computational model to explain the structure-activity relationship (SAR) and potentially guide their further optimization. The binding model here proposed places the benzopyran ring in the catalytic binding site, coordinating the Zn^2+^ ion through the oxygen in position 3, in contrast to previous hypothesis. The whole series of HFI compounds was then systematically simulated, starting from this binding mode, using molecular dynamics and binding affinity estimated with the linear interaction energy (LIE) method. The agreement with experimental affinities supports the binding mode proposed, which was further challenged by rigorous free energy perturbation (FEP) calculations. Here, we found excellent correlation between experimental and calculated binding affinity differences, both between selected compound pairs and also for recently reported experimental data concerning the site directed mutagenesis of residue Phe544. The computationally derived structure-activity relationship of the HFI series and the understanding of the involvement of Phe544 in the binding of this scaffold provide valuable information for further lead optimization of novel IRAP inhibitors.

## Introduction

Insulin-regulated aminopeptidase (IRAP, EC 3.4.11.3), also known as leucyl-cystinyl aminopeptidase, placental leucine aminopeptidase, and oxytocinase is a transmembrane zinc metalloenzyme that belongs to the M1 family of aminopeptidases (Lew et al., 2003). High levels of IRAP expression are found in areas of the brain associated with cognitive function including the hippocampus (Harding et al., 1992; Roberts et al., 1995; Chai et al., 2000), and it has been associated with several biological functions such as antigenic peptide processing for MHC Class I cross-presentation (Saveanu et al., 2009; Segura et al., 2009), GLUT4 regulation and transportation (Albiston et al., 2007), and regulation of oxytocin and vasopressin levels in the brain (Chai et al., 2004; Stragier et al., 2008; Hattori and Tsujimoto, 2013). In 2001, angiotensin IV (Ang IV) was identified as a potential inhibitor of IRAP (Albiston et al., 2001) along with LVV-hemorphin-7 (LVVYPWTQRF, a degradation product of β-globin) (Lee et al., 2003; Lew et al., 2003), both with *K*_*i*_ values in the nanomolar range.

**FIGURE 1:**
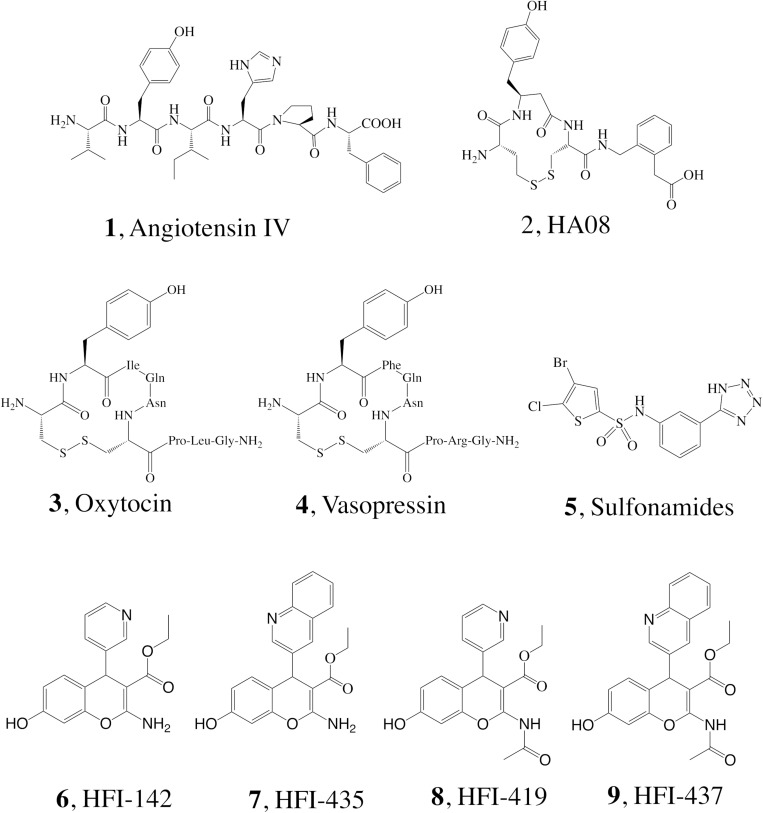
Structure of IRAP inhibitors and substrates.

It has been shown that inhibiting IRAP with Ang IV (1, Figure 1) and other structurally related peptidomimetics like HA08 (2) (Diwakarla et al., 2016b) is linked with improved memory and learning *in vivo* (Braszko et al., 1988; Wright et al., 1993, 1996, 1999; O’Malley et al., 1998; De Bundel et al., 2009; Fu et al., 2012), including enhancement of dendritic spine density (DSD) exerted by HA08 in hippocampal cells (O’Malley et al., 1998; Fu et al., 2012), as well as drug mitigation and lesion-induced memory deficits in rodents (Vauquelin et al., 2002; Albiston et al., 2003; Chai et al., 2004). Endogenous IRAP substrates such as the macrocyclic peptides oxytocin (3) and vasopressin (4, Figure 1) also improve cognitive parameters in the brain (Chai et al., 2004; Stragier et al., 2008). Consequently, it is not surprising that during the last 10–15 years, considerable efforts have been devoted to the discovery of small molecule IRAP inhibitors as potential cognitive enhancers. Comprehensive reviews are now available, and existing IRAP inhibitors reported include drug-like scaffolds like sulfonamides (5) or benzopyrans (6–9, Figure 1) (Hallberg, 2009; Barlow and Thompson, 2020; Georgiadis et al., 2020; Hallberg and Larhed, 2020). The later scaffold was identified in 2008 by virtual screening, and subsequently optimized resulting in a series coined as HFI (Howard Florey Institute) (Albiston et al., 2008). The most potent inhibitors present affinity values within the nanomolar range, and include either a 4-(pyridin-3-yl) or a 4-(isoquinolin-3-yl) substituent at the benzopyran and also a 2-amino or 2-acetamido substitution (Figure 1; Albiston et al., 2008). Recently it was demonstrated that HFI compounds, exemplified by HFI-419 (8), enhance spatial working memory possibly by promoting the formation of functional dendritic spines by facilitating GLUT4-mediated glucose uptake into hippocampal neurons (Seyer et al., 2020).

A binding mode and derived SAR of the inhibitory mechanism of the HFI series was initially proposed on the basis of a homology model of the catalytic domain of IRAP, which was built on the template of the equivalent domain of leukotriene A4 hydrolase (E.C. 3.3.2.6; LTA4H, PDB ID: 1HS6) (Thunnissen et al., 2001). Benzopyrans are chiral molecules, and the model proposed presented the *S-*isomer as the active enantiomer, with two well defined binding poses for pyridinyl and the quinolinyl derivatives, respectively (Albiston et al., 2010); while the pyridinyl derivatives would coordinate the Zn^2+^ ion through the benzopyran oxygen, for the quinolinyl compounds the coordination was predicted to occur through the nitrogen in this ring. Based on this model, a full series of HFI compounds was synthesized and pharmacologically characterized in 2014 (Mountford et al., 2014). However, the crystal structures of IRAP reveal a more open binding crevice as compared to other aminopeptidases. In the first crystal structure of IRAP (PDB code 4PJ6), the particular conformation of the GAMEN loop was related to the IRAP specificity for hydrolyzing cyclic peptides (Hermans et al., 2015). Even the last crystal structure of IRAP with the macrocyclic inhibitor HA08 (2), reported during the preparation of this manuscript, shows a binding site more open than in LTA4H, since the partially closed GAMEN loop is compensated with a rearrangement of the topology of some helical domains (Mpakali et al., 2020).

A computational docking study of HFI compounds performed right after the release of the first IRAP crystal structure suggested this time the *R-*enantiomer as the bioactive one, showing a unified binding orientation across the whole series in contrast to the previous models. In this new modeling, the Zn^2+^ ion would be coordinated by the oxygen of the hydroxyl group from the benzopyran ring, while the chromene ring remained packed against the GAMEN loop (Hermans et al., 2015). A later crystal structure of the closed conformation of IRAP (PDB ID: 5MJ6), was recently used to propose a different binding pose for compound 9 (HFI-437) (Mpakali et al., 2017). However, none of the binding modes proposed so far could explain the SAR of the HFI series, which motivated the present study. In addition to the SAR, we were interested in the role of specific residues in ligand binding. To accomplish these goals, we designed an approach combining molecular docking, molecular dynamics (MD) and binding free energy calculations utilizing both linear interaction energy (LIE) and free energy perturbation (FEP) approaches. These models were analyzed on their capacity to reproduce and explain the available experimental affinity data of the HFI inhibitor series for the wild type (WT) IRAP (Mountford et al., 2014), as well as the mutagenesis data of position Phe544 for a subseries of HFI compounds (Albiston et al., 2010). Our results suggest a unified binding mode that is compatible with all the (SAR) data available for this series, allowing a better understanding of the molecular details involved in inhibitor binding to IRAP.

## Materials and Methods

### Preparation of the IRAP Structure and Ligand Docking

The crystal structure of human IRAP was retrieved from the protein data bank (PDB code 4PJ6) (Hermans et al., 2015), and monomer A from the crystal dimer was retained for docking and molecular dynamics (MD) simulations. The structure was prepared with the *protein preparation wizard* utility in Maestro v. 9.2. (Schrödinger, LLC; NY, United States), involving addition of hydrogens and rotamer assignment of Asn, Gln, and His sidechains to optimize the H-bonding pattern. The F544I and F544V mutants were model on this structure using the Prime tool in Maestro, which allows adapting side chain conformation for neighboring residues to the modeled mutation. The 3D structures of all ligands (6–9, 15a-g, 16–18, see Figure 1 and Table 1) were built in Maestro and prepared with the LigPrep utility, which includes hydrogen addition considering most probable tautomers and isoelectric species and generation of independent stereoisomers, with a final optimization of the 3D structure. Docking explorations of subset 6–9 were performed with GLIDE-XP (Halgren et al., 2004) on a 30 Å cubic grid centered on the equivalent position of the Cα atom of His in Ang IV (Diwakarla et al., 2016b). These settings were applied in two separate docking approaches: (i) without constraints, and (ii) with constraints, where H-bond/Metal and Metal Coordination constraints against the Zn^2+^ ion were used in the receptor grid generation, to ensure a metal–ligand interaction preserving the initial metal coordination. Once one binding mode was determined for this subset, it was used as a template to dock the entire HFI-series of compounds (15a-g, 16a-r, 17a-g, 18d-h, see Table 1) in analogous orientation on the wt-IRAP using the Flexible Ligand Alignment in Maestro v. 9.2. All docking poses resulted from the docking calculations were retained for further analysis and MD calculations.

**TABLE 1:** LIE-Calculated and experimental binding free energies of HFI-series compounds.

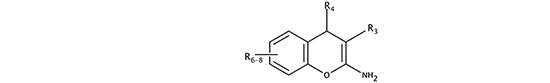						
**Compound**	**R_3_**	**R_4_**	**R_6–8_**	***K*_*i*_ (μM)^*a*^**	**Δ*G* Calc^*b*^**	**Δ*G* Exp^*c*^**
15a	CN	4-methoxyphenyl	7-OH	>100	−5.26 ± 0.12	>−5.45
15b	CN	3-methoxyphenyl	7-OH	>100	−5.35 ± 0.11	>−5.45
15c	CN	3,4-dimethoxyphenyl	7-OH	50	−5.56 ± 0.12	−5.86
15d	CN	3,5-dimethoxyphenyl	7-OH	>100	−5.83 ± 0.14	>−5.45
15e	CN	3,4,5-trimethoxypheny	7-OH	>100	−6.05 ± 0.17	>−5.45
15f	CN	pyridin-3-yl	7-OH	>100	−5.03 ± 0.26	>−5.45
15g	CN	4-N,N-dimethylaminophenyl	7-OH	>100	−5.40 ± 0.14	>−5.45
16a	CO_2_Et	phenyl	7-OH	>100	−5.46 ± 0.17	>−5.45
16b	CO_2_Et	2-cyanophenyl	7-OH	>100	−5.61 ± 0.19	>−5.45
16c	CO_2_Et	pyridin-2-yl	7-OH	2.9	−6.67 ± 0.19	−7.55
16d	CO_2_Et	quinolin-2-yl	7-OH	3	−7.64 ± 0.09	−7.53
16e	CO_2_Et	2-nitrophenyl	7-OH	42	−7.01 ± 0.08	−5.96
16f	CO_2_Et	3-chlorophenyl	7-OH	35	−7.15 ± 0.14	−6.07
16g	CO_2_Et	3-cyanophenyl	7-OH	3.2	−7.37 ± 0.11	−7.49
16h	CO_2_Et	2,4-dichloropyridine-3-yl	7-OH	14	−7.38 ± 0.18	−6.61
16i	CO_2_Et	4-methylphenyl	7-OH	>100	−5.24 ± 0.10	>−5.45
16j	CO_2_Et	4-bromophenyl	7-OH	>100	−6.00 ± 0.11	>−5.45
16k	CO_2_Et	4-chlorophenyl	7-OH	>100	−5.86 ± 0.16	>−5.45
16l	CO_2_Et	4-cyanophenyl	7-OH	11	−7.55 ± 0.08	−6.76
16m	CO_2_Et	pyridin-4-yl	7-OH	3.7	−7.23 ± 0.07	−7.40
16n	CO_2_Et	quinolin-4-yl	7-OH	0.9	−7.93 ± 0.17	−8.24
16o	CO_2_Et	4-nitrophenyl	7-OH	7.7	−7.83 ± 0.22	−6.97
16p	CO_2_Et	4-(pyridin-2-yl)phenyl	7-OH	>100	−5.96 ± 0.18	>−5.45
16q	CO_2_Et	4-N,N-dimethylaminophenyl	7-OH	5.3	−7.57 ± 0.20	−7.19
16r	CO_2_Et	3,4-dimethoxypheny	7-OH	6.2	−7.72 ± 0.21	−7.10
17a	CO_2_Me	pyridin-3-yl	7-OH	4.9	−6.86 ± 0.07	−7.24
17b	CO_2_Pr	pyridin-3-yl	7-OH	1.6	−7.13 ± 0.14	−7.90
17c	CO_2_n-But	pyridin-3-yl	7-OH	2.6	−7.07 ± 0.17	−7.61
17d	CO_2_t-But	pyridin-3-yl	7-OH	11.9	−7.15 ± 0.15	−6.71
17e	CO_2_-(CH_2_)_2_-O-CH_3_	pyridin-3-yl	7-OH	4	−6.97 ± 0.20	−7.36
17g	CO_2_-Bz	pyridin-3-yl	7-OH	1.7	−7.31 ± 0.08	−7.86
18d	CO_2_Et	pyridin-3-yl	6-Cl, 7-OH	5.6	−7.33 ± 0.24	−7.16
18f	CO_2_Et	pyridin-3-yl	8-OH	9.8	−6.79 ± 0.21	−6.83
18g	CO_2_Et	pyridin-3-yl	7-8, fused phenyl	>100	−7.47 ± 0.10	>−5.45
18h	CO_2_Et	pyridin-3-yl	5-6, fused phenyl	>100	−5.49 ± 0.21	>−5.45

*^*a*^Experimental data extracted fromMountford et al. (2014). ^*b*^Energies in kcal mol**^–^**^1^, obtained from the optimized LIE model, with α = 0.285, β = 0.138, γ = −1.2 and expressed as mean ± 1 SEM from the replicate simulations. Average unsigned error is 0.5 kcal mol**^–^**^1^ (considering only compounds with experimentally determined *K*_*i*_). ^*c*^Experimental values obtained as $\Delta \,G_{b\,i\,n\,d}^{e\,x\,p}\,R\,T\,l\,n\,K_{i}$.*

### Molecular Dynamics (MD) Simulations

Molecular dynamic simulations were performed with spherical boundary conditions using the program Q (Marelius et al., 1998), optimized for efficient sampling suitable for the free energy calculations for ligand series or protein mutations (Boukharta et al., 2014; Shamsudin Khan et al., 2015). The OPLS-AA force field for proteins was used (Jorgensen et al., 1996), in combination with compatible parameters for Zn^2+^ ion and automatic parametrization of the ligands within the same force field, performed with Macromodel version 10.6 (Schrödinger, LLC; NY, United States). A simulation sphere of 25 Å radius centered on the equivalent position of the Cα atom of His4 in Ang IV was built as previously described (Diwakarla et al., 2016a,b; Vanga et al., 2018). The sphere was solvated with TIP3P water molecules (Jorgensen et al., 1983) and subjected to polarization and radial constraints according to the surface constrained all-atom solvent (SCAAS) model (King and Warshel, 1989; Marelius et al., 1998) to mimic the properties of bulk water at the sphere surface. Protein atoms outside the simulation sphere were restrained to their initial positions and only interacted with the system through bonds, angles, and torsions. Excluding His, all other titratable residues within 20 Å of the Zn^2+^ ion were treated in their charged form. In addition, the residues Lys520, Lys726, Glu767, Asp773, Arg817, Glu818, Arg820, Glu825, Arg858, Glu887, Lys890, Lys892, Glu895, Arg933, and Glu1002, within the 20–25 Å layer of the sphere were also treated as ionized since they are forming salt bridges, while all other ionizable residues within this layer were treated as neutral to avoid insufficient dielectrical screening. With this setup, the simulation sphere was overall neutral, thus avoiding the consideration of additional Born terms in the calculation of free energies of charged ligands as compared to bulk solvent. Non-bonded interactions were calculated explicitly up to a 10 Å cutoff, except for the ligand atoms for which no cutoff was used. Beyond the direct cutoff, long-range electrostatics were treated with the local reaction field (LRF) multipole expansion method (Lee and Warshel, 1992). During a 175 ps equilibration stage, the system was slowly heated to the target temperature of 310 K while initial positional restrains on all solute heavy atoms were gradually released. The subsequent data collection phase consisted of 10 replicate MD simulations of 2 ns each, with randomized initial velocities, accounting for a total of 20 ns sampling trajectories where the ligand-surrounding energies were collected for binding affinity calculations. A time step of 1 fs was used and no positional restraints were applied. Solvent bonds and angles were constrained using the SHAKE algorithm (Ryckaert et al., 1977). Non-bonded pair lists were updated every 25 steps, and the ligand-surrounding interaction energies were sampled every 50 steps. In order to estimate free energies of binding, the same setup was used for the reference state calculations. For the LIE and ligand-FEP simulations, this involves sampling the ligand-surrounding energies in parallel MD simulations of the ligand solvated in water, while for the residue FEP simulations the reference MD simulations involve the apo state of the protein, i.e., without the ligand complexed.

### Linear Interaction Energy (LIE) Calculations

Binding free energies for every compound were calculated for every docked ligand using the linear interaction energy (LIE) method as (Aqvist et al., 1994; Hansson et al., 1998)

(1) $$\Delta \,G_{b\,i\,n\,d}^{c\,a\,l\,c}=\alpha \,\Delta \,\left\langle U_{l-s}^{v\,d\,W}\right\rangle +\beta \,\Delta \,\left\langle U_{l-s}^{e\,l}\right\rangle +\gamma$$

where, $\Delta \,\left\langle U_{l-s}^{v\,d\,W}\right\rangle$ and $\Delta \,\left\langle U_{l-s}^{e\,l}\right\rangle$ are the differences in the average nonpolar and polar ligand-surrounding interaction energies in the two states, that is, water solvated (free ligand) and in complex with the protein (bound ligand). The coefficients α and β are scaling parameters (Hansson et al., 1998; Almlöf et al., 2004, 2007) for the nonpolar and polar terms, respectively. In the standard LIE model, α has a value of 0.18, while β depends on the chemical nature of the ligand. The IRAP active site has a divalent Zn^2+^ ion together with a cluster of carboxylates, causing very large electrostatic interaction energies with the ligands. Because these interaction energies, particularly those involving the Zn^2+^ ion, will be very sensitive to the force field parameters, we follow a protocol used earlier for binding sites containing ions (Mishra et al., 2012) and treated β as a free parameter. The reported non-bonded energies correspond to average values over 10 replicate MD simulations on each state (free and bound), and the corresponding errors are calculated as the standard error of the mean (SEM).

Experimental binding free energies $(\Delta G_{b\,i\,n\,d}^{e\,x\,p}$) were extracted from inhibition constant (*K*_*i*_) experimental values as

(2) $$\Delta \,G_{b\,i\,n\,d}^{e\,x\,p}=R\,T\,l\,n\,K_{i}$$

### Free Energy Perturbation (FEP) Simulations

#### Ligand FEP

The relative binding free energy between selected pairs of ligands, A and B, was calculated using the free energy perturbation (FEP) method. In this method ligand A is transformed into B in parallel MD simulations in both the protein-bound ligand and the free (water-solvated) reference state. The construction of a closed thermodynamic cycle connecting these processes allows the estimation of the relative binding free energy between the pair of ligands (ΔΔ*G*_*bind*,*hboxB*−*A*)_), as the difference in the free energy of each transformation of A → B, as

(3) $$\Delta \,G_{b\,i\,n\,d}^{B}-\Delta \,G_{b\,i\,n\,d}^{A}=\Delta \,\Delta \,G_{b\,i\,n\,d}=\Delta \,G_{b\,o\,u\,n\,d}^{A\to B}-\Delta \,G_{f\,r\,e\,e}^{A\to B}$$

The free energy difference associated with each ligand transformation was calculated using Zwanzig’s exponential formula (Zwanzig, 1954)

(4) $$\Delta \,G^{A\to B}=\Delta \,G_{B}-\Delta \,G_{A}=-\beta^{-1}\,\sum_{m=1}^{n-1}\text{ln}\,{\left\langle \text{exp}\,\left(-\beta^{-1}\,\left(U_{m+1}-U_{m}\right)\right)\right\rangle }_{m}$$

where *U_m* denotes the effective potential energy function of a particular FEP window and *n* is the number of intermediate states. *U_m* is constructed as a linear combination of the initial (A) and final (B) potentials

(5) $$U_{m}=\left(1-\lambda_{m}\right)\,U_{A}+\lambda_{m}\,U_{B}$$

where the coupling parameter λ_**m**_ is stepwise incremented from 0 to 1, in our case divided into 51 λ-windows, where every window is sampled for 30 ps.

#### Residue FEP

Similar to the ligand FEP method, relative binding free energies associated with amino acid side-chain mutations are calculated following Eq. 4–6, but instead the two states (A and B) correspond to the wild-type (wt) and mutant (mut) versions of the enzyme. This protocol is based on a computational alanine scanning protocol developed in our lab, which makes use of the additive property of thermodynamic cycles sharing a common leg to allow non-alanine mutations (Boukharta et al., 2014; Keränen et al., 2015). During annihilation of a side-chain, each atom group will undergo three consecutive transformations (i) annihilation of partial charges, (ii) introduction of a soft-core potential for the van der Waals (Lennard-Jones) potential to prevent singularities, and (iii) annihilation of the soft-core potential. Depending on the nature of the starting sidechain, and the number of atom-groups therein defined, this involves a number of stages that varies from 4 (for simple cases like Set to Ala) to 11 (i.e., Trp to Ala). Each of these stages is performed over a number of FEP windows (51 λ-windows, each sampled for 30 ps), assuring enough sampling to achieve converged results. The full transformation (wt → Ala) is performed in in 10 replica molecular dynamics (MD) simulations for each state, which in this case involves the receptor in complex with the ligand (*holo*) and the free (*apo*) receptor. The difference in ligand binding free energy between the wt and Ala (mut) versions of the receptor ($\Delta \,\Delta \,G_{b\,i\,n\,d}^{w\,t-a\,l\,a}$) can be estimated by solving the associated thermodynamic cycle. It follows that, for non-alanine mutations, one can start the same set of annihilations to Alanine starting from the modeled (mut) state, and calculate the associated ($\Delta \,\Delta \,G_{b\,i\,n\,d}^{m\,u\,t-a\,l\,a}$). The relative free energy associated with non-Ala mutations (in our case, F544I and F544V) will be the result of the combination of the corresponding thermodynamic cycles as

(7) $$\Delta \,\Delta \,\Delta \,G_{b\,i\,n\,d}^{m\,u\,t-w\,t}=\left(\Delta \,G_{h\,o\,l\,o}^{w\,t}-\Delta \,G_{a\,p\,o}^{w\,t}\right)-\left(\Delta \,G_{h\,o\,l\,o}^{m\,u\,t}-\Delta \,G_{a\,p\,o}^{m\,u\,t}\right)\,$$

When performing several mutations for the same position, the wt → Ala of the cycle needs to be calculated only once and can be reused for any mutation at that position. Hysteresis as a measure of convergence is calculated as the absolute difference between forward and reverse pathways of each subperturbation. The total hysteresis is the sum of the hysteresis values for each subperturbation of the transformation.

## Results

### Docking, MD and LIE Calculations

**FIGURE 2:**
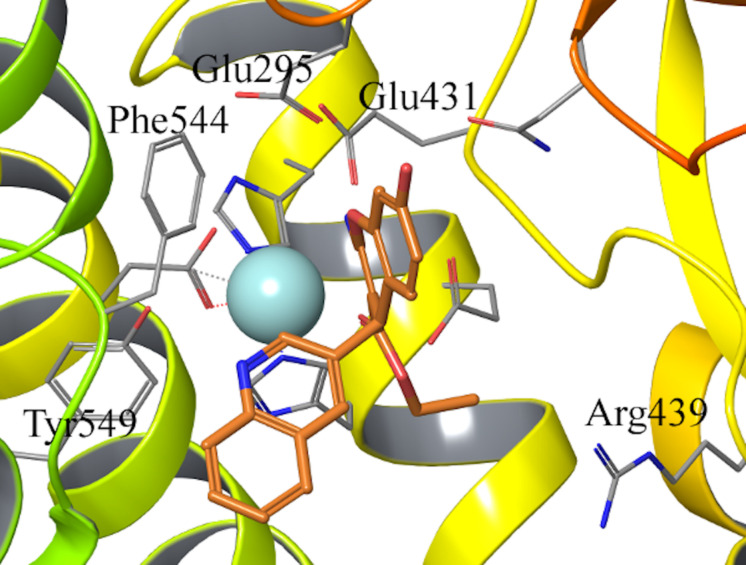
Binding pose of compound 7 (orange sticks) in IRAP active site obtained from the constrained docking calculations. Key residues in the active site are shown as gray lines, and the Zn^2+^ as light blue sphere.

The binding mode of compound 6–9 was explored with two independent docking protocols, and the poses obtained for each ligand were followed by MD relaxation. This stage allowed discarding any binding orientation that did not show structural stability. In particular, none of the binding poses obtained with the “no constraints” docking with GLIDE (Halgren et al., 2004) showed any direct contact with the Zn^2+^, despite being in the vicinity of the ion, and the simulations confirmed unstable binding modes in all cases, with protein-ligand interactions lost during the MD sampling. Conversely, the docking pose obtained for *R*-isomer of ligand 7 (HFI-435) by imposing an interaction with the cation as a constraint, revealed itself as a stable binding mode after the MD stage. This binding mode showed coordination to the Zn^2+^ through the ester carbonyl oxygen, while the benzopyran ring formed a stable π-stacking interaction with Phe544, accompanied by frequent polar interactions of the exocyclic 2-amino group with Glu431 and Glu295. The quinolone ring in R_4_ displayed π-stacking with Tyr549 (Figure 2). This binding mode was then retained and used as a template to build the entire HFI-series of compounds (Mountford et al., 2014) (15a-g, 16a-r, 17a-g, 18d-h, see Table 1) in analogous orientation on the wt-IRAP. Additionally, for ligands 6–9 we also generated the corresponding complex with the two IRAP mutants here investigated (F544I and F544V). The next step was the estimation of the ligand binding affinities to the wt-IRAP for the whole series. For this, the same MD sampling used in the previous stage was replicated 10 times per ligand-IRAP complex, accompanied by the same MD sampling of each ligand in a similar water sphere, all of which was used as a basis to calculate the binding free energy with the linear interaction energy (LIE) method (Aqvist et al., 1994).

**FIGURE 3:**
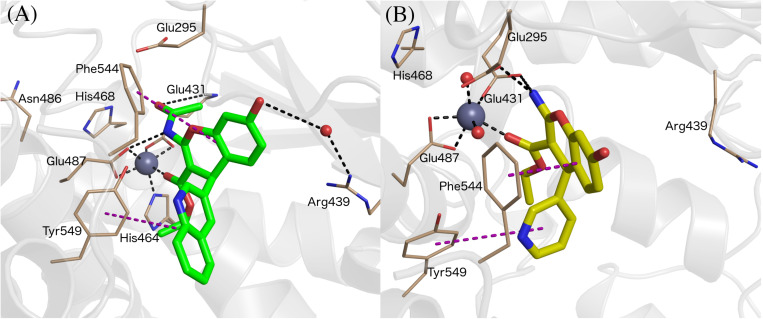
Binding mode of compound 9 (panel **A**, green sticks), and 6 (panel **B**, yellow sticks) in IRAP as determined from the MD simulations used for LIE calculations. Zn^2+^ and water molecules are shown as gray and red spheres, respectively. All of the figures shown are extracted from representative snapshots of 2 ns MD simulations. H-bonds are shown as black dotted line, and π – stacking interactions in the purple color dashed line.

All compounds were stable during these MD simulations, maintaining the key coordination of the Zn^2+^ through the carbonyl oxygen of the 3-ethyl ester, with the exception of the 3-cyano derivatives 15a-g. For these compounds, it was found that the 3-cyano group could not be accommodated near the metal while preserving other key protein-ligand interactions, resulting in instability of the initial binding pose (see Supplementary Table 1, indicating average ligand RMSD along the MD trajectories) and consequently low values for the LIE estimated binding free energies, due to loss of key interactions with IRAP. This observation is in line with the experimental data, showing ligands 15a-g as inactive compounds (only 15c has measured *K*_*i*_, but as low as 50 μM). Compounds 8 and 9 showed an edge to face π-stacking interactions with Phe544 (Figure 3A). In these compounds, the hydroxyl group at the position 7 of the benzopyran ring interacts with Arg439 via a water molecule, while Tyr549 makes π-stacking with the 3-pyridyl (8) or 3-quinolinyl (9) rings (Figure 3A). In compounds 7 and 8, the central core ring is stabilized by π-stacking interactions with Phe544 (parallel π-stacking) and Tyr549 (edge-to-edge), while the free 2-amino group makes H-bonds with Glu295 and Glu431. Interestingly, along the MD simulations of all 2-amino compounds the role of His464 and His468 in coordination of the Zn^2+^ ion was replaced with two water molecules (Figure 3B).

Owing to the strong electrostatic ligand interactions with the Zn^2+^ ion, which is very sensitive to given force field parameters, it was found necessary in previous LIE calculations to determine the parameters α, β, and γ (Diwakarla et al., 2016a,b; Vanga et al., 2018) empirically. The earlier derived LIE parameters were based on a few chemically diverse compounds. In the present series, 25 out of 39 compounds have experimentally determined *K*_*i*_ values for the wild-type enzyme, while the remaining compounds (non-binders) have *K*_*i*_ values > 100 μM. Hence, we selected this subset of 25 compounds to optimize the LIE model by independent fitting. We applied the resulting LIE parameterization to estimate the affinities to wt-IRAP for the entire series, including the known non-binders. The results, summarized in Table 1, show excellent agreement with the experimental data, with a calculated mean unassigned error (MUE) for the 25 compounds with measured *K*_*i*_ as low as was 0.50 kcal/mol.

For compounds 6–9, binding free energies were estimated not only with wt-IRAP, but also with the F544I and F544V modeled mutants, for which experimental data was available (Albiston et al., 2010). Replacement of Phe544 with either Ile or Val led to the loss of the π- stacking between the wt sidechain and the benzopiran, partially explaining the significant decline in binding affinity observed in most cases (see Table 2). Indeed, the correlation of the LIE-calculated with the experimental values for the mutations on these four ligands was quite remarkable (Table 2), with a calculated MUE of 0.5, 0.68, and 0.5 kcal/mol for wt-IRAP, F544I, and F544V, respectively.

**TABLE 2:** LIE calculated and experimentally binding free energies (ΔG, in kcal/mol) for compounds 6–9 in wild type and mutant IRAP.

	**Δ*G* (kcal mol^–1^)^*a*^**	
**Compound**	**LIE^*b*^**	**Experimental^*c*^**
	**wt-IRAP**		
6	−8.17 ± 0.4	−7.76 ± 0.26
7	−8.73 ± 0.4	−8.83 ± 0.43
8	−8.56 ± 0.1	−8.35 ± 0.41
9	−9.09 ± 0.2	−10.23 ± 0.13
	
	**F544I**		
6	−7.71 ± 0.4	−6.52 ± 0.33
7	−7.29 ± 0.1	−7.40 ± 0.17
8	−7.53 ± 0.1	−7.82 ± 0.19
9	−8.09 ± 0.2	−9.13 ± 0.15
	
	**F544V**	
6	−6.61 ± 0.4	−7.34 ± 0.02
7	−7.54 ± 0.2	−7.63 ± 0.21
8	−8.26 ± 0.1	−8.41 ± 0.20
9	−8.34 ± 0.2	−8.95 ± 0.30

*^*a*^Energies are expressed as mean ± S.E.M. estimated from independent experiments (Albiston et al., 2010) or replicate MD simulations (LIE). ^*b*^Optimized LIE model, with α = 0.285, β = 0.138, γ = −1.2. ^*c*^Experimental values are extracted from Albiston et al. (2010).*

**FIGURE 4:**
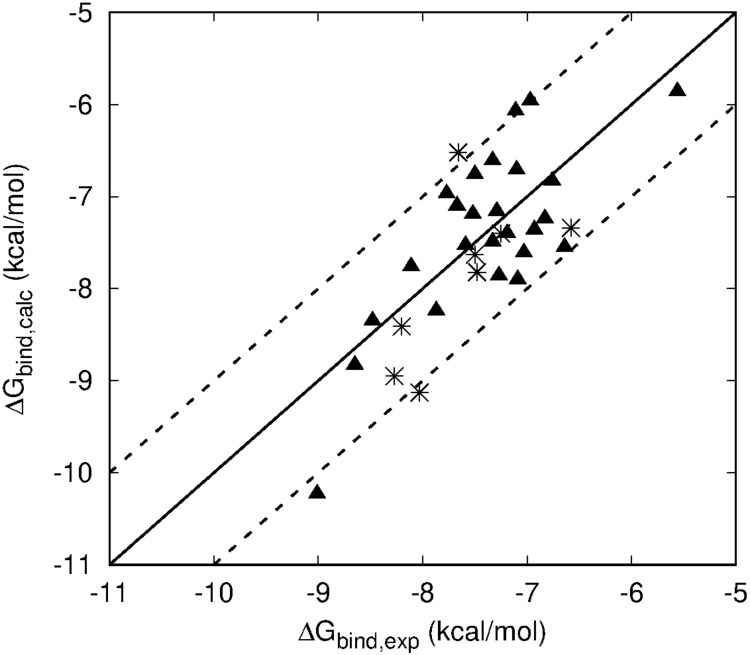
Scattered plot of LIE calculated (*Y* axis) and experimental (*X*-axis) binding affinities of all HFI compounds that have experimentally determined *K*_*i*_ values for WT (triangles) and F544I/V mutant version (stars) of IRAP. Main line denotes perfect agreement with experiments, while the two dashed lines are +/– 1 kcal⋅mol^– 1^.

The overall results of the LIE model are summarized in Figure 4, showing the correlation between calculated and experimental binding free affinities for the HFI compounds with experimental *K*_*i*_ values for both wt (triangles) and mutants (stars). This model not only shows very small average deviations from experimental values (overall MUE = 0.51 kcal/mol), but the relative ranking within the series, which is key to assess further optimization efforts, is excellent with a Pearson correlation coefficient, *R* = 0.71.

### Free Energy Perturbation Calculations

**FIGURE 5:**
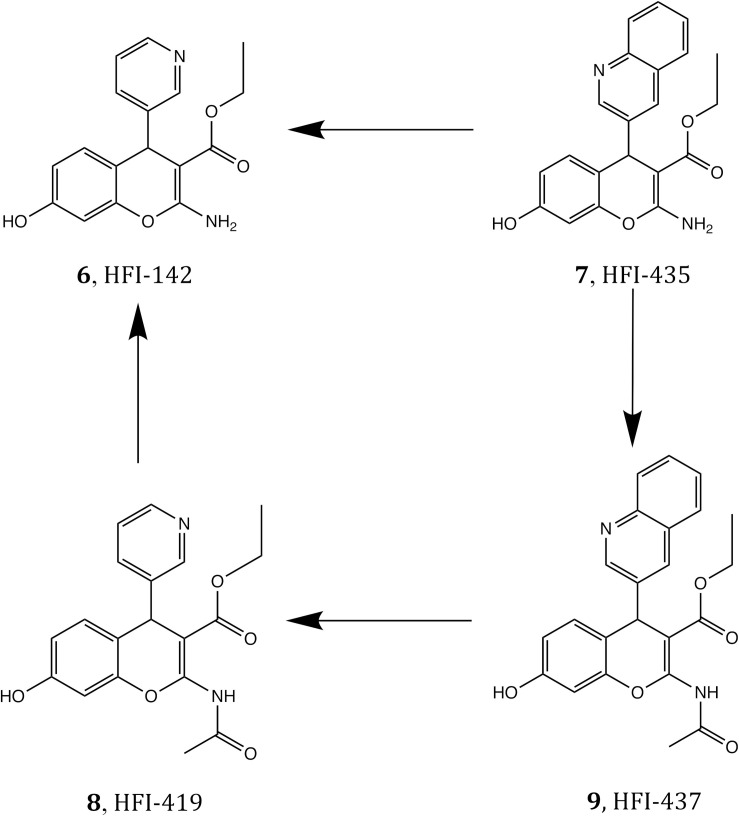
Closed thermodynamic cycle depicting the FEP pair transformations that connect the four compounds 6–9.

To further confirm the binding mode proposed for the HFI series and the derived SAR, we designed a set of free energy perturbation (FEP) transformations between selected pairs of ligands, following the experimental design illustrated in Figure 5. The advantage of this approach is that, by estimation of the relative binding free energies (ΔΔ*G*) between four pairs of compounds, one can not only compare with the corresponding experimental differences in affinity, but also estimate the error of such calculations, since the overall energy change along the closed thermodynamic cycle is Δ*G* = 0 kcal/mol. The vertical legs of the closed thermodynamic cycle shown in Figure 5 explore the effect of replacing the acetamide by a free amino group in position 2 of the benzopyran ring, while the horizontal legs refer to the change of replacing 3-quinoline by 3-pyridyl.

**FIGURE 6:**
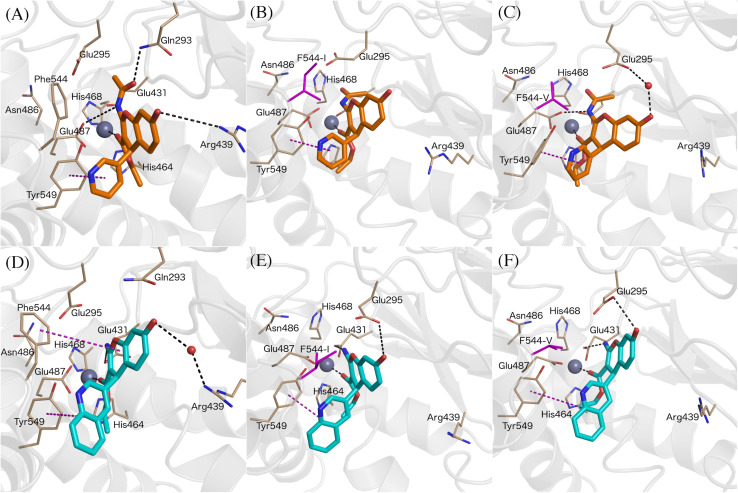
Binding modes of compound 8 (**A**, orange sticks) and 7 (**D**, cyan sticks) in wt-IRAP active site determined by FEP simulations. Binding mode of compound 8 in F544I **(B)** and F544V **(C)**. Binding mode of compound 7 in F544I **(E)** and F544V **(F)**. All of the figures shown are extracted from representative snapshot simulations. Residues playing a role in ligand binding are explicitly shown, while residues F544I and F544V are represented in magenta color. The remaining atom representations and protein-ligand interactions following the scheme as in Figure 3.

The results of these FEP simulations are summarized in Table 3 and Figure 6. The detrimental effect of replacing the acetamide by a free amino group in position 2 of the benzopyran ring was observed in both 9 → 7 and 8 → 6 FEP transformations. Our model reproduces the experimental effect very accurately, and assigns this effect to the loss of the interaction established between acetamide oxygen with the Glu431 backbone (Figure 6D). Interestingly, the detrimental effect of replacing 3-quinoline by 3-pyridyl (transformation of 9 → 8 and 7 → 6) was indirect, inducing the loss of the π-stacking interactions between the ligand and Phe544, while no significant effect was observed in the region of the substituent changed. During the transformation 9 → 8, these changes were accompanied by a replacement of the interaction of the acetamide oxygen with the backbone of Glu431, to establish a new interaction with Gln293, as shown in Figure 6A. These structural rearrangements explain the large experimental shifts in relative binding affinities between compounds 9 and 8, which were captured in our FEP simulations (Table 3). Overall, the calculated relative affinity change between the four compound pairs is in excellent agreement with the experimental data, with a MUE = 0.04 kcal/mol and an equally negligible cycle closure error of 0.09 kcal/mol, reinforcing the ability of this binding mode to explain the SAR of this series.

**TABLE 3:** Experimental and FEP calculated relative binding free energies between ligand within the subset of compounds 6–9, both in wild type and mutant versions of IRAP.

**Transformation**	**ΔΔ*G* (B – A, kcal mol^–1^)^*a*^**	
**A → B**	**FEP**	**Experimental^*b*^**
	**wt-IRAP**		
8→6	0.68 ± 0.70		0.58 ± 0.34
7→6	1.10 ± 0.46		1.07 ± 0.36
9→8	1.90 ± 0.18		1.89 ± 0.30
9→7	1.38 ± 0.50		1.40 ± 0.32
	
	**F544I–IRAP**		
8→6	1.44 ± 0.98		1.30 ± 0.27
7→6	0.87 ± 0.29		0.88 ± 0.26
9→8	1.39 ± 0.20		1.31 ± 0.17
9→7	0.74 ± 0.37		1.73 ± 0.16
	
	**F544V–IRAP**		
8→6	1.04 ± 0.65		1.07 ± 0.14
7→6	0.61 ± 0.24		0.29 ± 0.15
9→8	0.78 ± 0.30		0.54 ± 0.26
9→7	1.40 ± 0.25		1.32 ± 0.26

*^*a*^Energies are expressed as mean ± S.E.M. estimated from independent experiments (Albiston et al., 2010) or replicate MD simulations (FEP). ^*b*^ Experimental energies obtained from *K*_*i*_ values (Albiston et al., 2010) using the relation $\Delta \,\Delta \,G_{b\,i\,n\,d,e\,x\,p}^{o}\,R\,T\,l\,n\,\left(\frac{K_{i}\,\left(B\right)}{K_{i}\,\left(A\right)}\right).$*

The next question was to evaluate the mutagenesis effects with FEP simulations. A first approach was to perform the four transformations illustrated in Figure 5 but considering the F544I and F544V IRAP mutants. While the four ligands bound in a conserved way as compared to the wt-IRAP, several important interactions are missing. For example, as shown in Figure 6B, compound 8 shows only two direct interactions, with Zn^2+^ and a π- stacking with Tyr549. The experimental reduction in binding affinity can thus be assigned to the loss of critical interactions such as π-stacking with Phe544 and H-bonds with Arg439. The obtained relative binding free energies from the FEP transformations in the mutant versions of IRAP are again in excellent agreement with the experimental data (Table 3), with MUE values of 0.31 kcal/mol (F544I) and 0.17 kcal/mol (F544V), and calculated cycle closure errors of 1.22 (F544I) and −0.19 (F544V). The larger error observed for the F544I simulations is found to arise from the 9→7 transformation, which is off by about 1 kcal/mol from the experimental value.

The above calculations were further complemented with a direct simulation of the Phe544 mutation both to Val and Ile. The protocol to perform such *in silico* site-directed mutagenesis approach has been recently automated (Jespers et al., 2019) and thoroughly applied in our lab to characterize the site-directed mutagenesis effects on ligand binding for a number of GPCRs (Boukharta et al., 2014; Keränen et al., 2014, 2015). Here, one needs to perform the residue transformation both in the presence and in the absence of the bound ligand to fulfill the thermodynamic cycle, obtaining an estimated shift in the binding affinity of the ligand involved, due to the mutation examined. The FEP protocol is based on successive annihilation of the sidechain atoms to the common intermediate of alanine, which is performed once from the wt sidechain, and in parallel simulations for each of the modeled mutant sidechains, so that the two thermodynamic cycles can be joined and the total effect of wt → mutant calculated. The results, summarized in Table 4, show again excellent agreement with the experimental mutagenesis data, with MUE of 0.17 and 0.18 kcal/mol for Phe544Ile and Phe544Val, respectively, further confirming the validity of the proposed binding mode. It can be appreciated that for the case of the Phe → Ile transformation, the effect on ligand binding was more accurately described by this approach (i.e., *in silico* mutagenesis) rather than comparing the ligand transformations performed in wt and mutant versions of the enzyme. As opposed to the end-point LIE simulations, the FEP simulations show a maintained interaction between the ligand and Arg439 along the wt-IRAP transformations (8→ 6 and 9→ 7, Figures 6A,D). The fact that this interaction is selectively lost in the simulations on the two IRAP mutants provides a molecular mechanism to the reduced affinity besides the weakened interactions with the mutant (Val or Ile) sidechains replacing Phe544. The simulations also allowed to envisage the reasons of high affinity of compound 9, which when transformed to either compound 8 or 7, consistently loses interactions with Glu431 and Glu487 or with Glu295, respectively, on both mutant or the mutant versions of IRAP (Figure 6).

**TABLE 4:** Experimental and FEP calculated shifts in binding free energies due to point mutations of IRAP, for the subset of compounds 6–9. ΔΔ*G* (mut – WT, kcal mol^–^**^1^**)**^*a*^**.

	**ΔΔ*G* (mut – WT, kcal mol^–1^)^*a*^**						
	**F544A**	**I544A**	**V544A**	**F544I**		**F544V**	
	
**Compd**	**FEP**			**FEP**	***exp***	**FEP**	***exp***
6	0.93 ± 0.20	−0.35 ± 0.19	0.41 ± 0.18	1.28 ± 0.19	*1.24 ± 0.29*	0.52 ± 0.19	*0.42 ± 0.18*
7	0.92 ± 0.20	−0.73 ± 0.20	−0.59 ± 0.29	1.66 ± 0.20	*1.43 ± 0.33*	1.51 ± 0.25	*1.20 ± 0.34*
8	0.31 ± 0.17	−0.21 ± 0.21	0.36 ± 0.11	0.52 ± 0.19	*0.53 ± 0.32*	−0.06 ± 0.15	−*0.07 ± 0.32*
9	1.10 ± 0.22	−0.41 ± 0.18	−0.48 ± 0.15	1.51 ± 0.20	*1.10 ± 0.14*	1.58 ± 0.19	*1.28 ± 0.23*

*^*a*^Energies are expressed as mean ± S.E.M. estimated from independent experiments (Albiston et al., 2010) or replicate MD simulations (FEP).*

## Discussion

**FIGURE 7:**
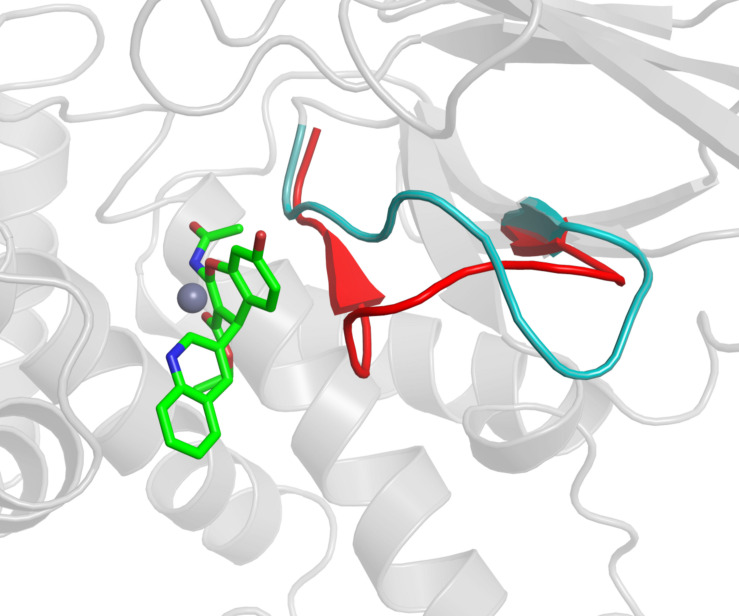
The GAMEN loop in the closed conformation (red color cartoon, PDB ID: 5MJ6) is compared with the GAMEN loop of open conformation (cyan color cartoon, PDB ID: 4PJ6) while compound 9 (green sticks) is docked in the active site. Zn^2+^ ion shown as gray sphere.

The benzopyran-based small molecule IRAP inhibitors (HFI series) constitute a promising chemotype for the development of first-in-class drugs for dementia and related diseases. Understanding the molecular mechanism of IRAP inhibition is thus crucial for the further hit-to-lead and lead optimization process of the existing collection of HFI compounds. In the absence of a crystal structure of any IRAP-HFI complex, we conducted an exhaustive docking study on the most potent compounds using the crystal structure of the semi-open conformation of IRAP (PDB 4PJ6). The selection of this conformation of the enzyme was based on the fact that it presents a different orientation of the GAMEN loop, as compared to the latest closed conformation structure of IRAP (PDB ID: 5MJ6). The superposition of the HFI-complex here determined with the closed conformation of IRAP, depicted in Figure 7, shows, however, no predicted steric clashes with the closed configuration of the GAMEN loop, thus suggesting that the HFI-series of compounds may bind to different IRAP conformations. The validity of the binding pose was further assessed by systematic estimation of the binding affinity for the complete series with the LIE approach, showing excellent agreement with the experimental data (*R* = 0.71, MUE = 0.5 Kcal/mol). More important, this binding mode allowed for a consistent interpretation of the full SAR for this series, in contrast with previous proposals in the literature. Such binding poses were proposed based on homology model (Albiston et al., 2010), semi-open (Hermans et al., 2015), and closed conformations (Mpakali et al., 2017) of IRAP, but all of them failed in explaining some intriguing features of this series. One particularly intriguing aspect not solved in the previous models was the role of 3-position substitutions on the chromene ring and the inactivity of 3-cyano compounds. Our binding pose of the HFI chemotype can explain this inactive series by highlighting the role of the oxygen of the ester group in position 3 in coordinating the Zn^2+^ ion. Thus, the 3-cyano series (15a-g) show instability of the predicted binding pose (high ligand RMSD values as compared to the compounds with measurable affinity), losing the key interactions with the enzyme and consequently the trajectories resulted in low LIE predicted free energies of binding. The LIE model also captures the observed tolerance for the aliphatic chain of the ester, even admitting an aromatic chain (17g) once the substituent at position 4 is fixed on the pyridine-3-yl substituent (series 17a-g, Table 2). Finally, the role of the hydroxy substituent in the chromone scaffold is also tuned down in our model, presenting this group toward a solvent-exposed area with polar residues in the vicinity, in agreement with the experimental SAR showing that this substituent can be swapped between positions 7 and 8 of the benzopyran ring (18f) while a fused phenyl ring is absolutely detrimental for the affinity (compounds 18g, 18h).

One common aspect with previous binding models is the role of residue Phe544, an important anchoring point through π – stacking with the benzopyran ring (Figure 3). The experimental mutagenesis data on this residue (Albiston et al., 2010) was here computed in different ways, providing further support for this interaction but quantifying its effect. LIE simulations reveal that the interactions with the Zn^2+^ are maintained in the Phe544 mutant versions, explaining the moderate effect of mutation of this position to hydrophobic sidechains. However, moderate changes in affinity due to marked structural changes, as is a sidechain mutation or the type of the chemical modifications within series 6–9 (topological changes on a ring, amide versus substituted acetamide) might fall out of the sensibility of LIE modeling. For this reason, we investigated the 6–9 series and the mutagenesis effects with FEP simulations. The use of complementary FEP transformations (i.e., ligand perturbations on different enzyme forms, and receptor mutation upon binding of different ligands) provides “two sides of the same coin” that provide a comprehensive perspective of the ligand binding process, as recently showed for the elucidation of the binding mode of A_2A_ adenosine receptor antagonists (Jespers et al., 2020). In the IRAP system, these simulations allowed to identify interactions that were selectively lost on the mutant versions of the enzyme (i.e., between the ligand and Arg439), or differences in ligand binding consistently observed within wt and mutant versions of the enzyme, as the case of the interactions of compound 9 with the network of glutamic acid residues (Figure 6). These simulations also showed the role of residues like Tyr549 and Glu295 and Glu431, suggesting further site directed mutagenesis experiments to probe this model.

In summary, the binding model for the HFI series here presented, which is supported by different methods of free energy calculations, provides a unified model across the series that satisfactorily explains the observed SAR of the series. Moreover, this binding model points to a relatively promiscuity for the conformations of IRAP, and sets the grounds for further structure-based optimization.

## Data Availability Statement

The raw data supporting the conclusions of this article will be made available by the authors, without undue reservation.

## Author Contributions

SV designed, performed and analyzed the experiments, and wrote the first draft. HG-D-T designed and analyzed the experiments and wrote the manuscript. JÅ and AH designed the experiments and contributed to the final writing of the manuscript. All authors contributed to the article and approved the submitted version.

## Conflict of Interest

The authors declare that the research was conducted in the absence of any commercial or financial relationships that could be construed as a potential conflict of interest.
