# Supplementary material for: Structural Basis of Inhibition of Human Insulin-Regulated Aminopeptidase (IRAP) by Benzopyran-Based Inhibitors
Source: Front Mol Biosci. 2021 Apr 1;8:625274. doi: 10.3389/fmolb.2021.625274 (PMC8047434; doi:10.3389/fmolb.2021.625274)
Supplement: Supplementary file 1 [file Table_1.pdf]

frontiers

Supplementary Material

Structural Basis of Inhibition of Human Insulin-Regulated Aminopeptidase (IRAP) by benzopyran-based inhibitors

Sudarsana Reddy Vanga[1], Johan Åqvist[1], Anders Hallberg[3], Hugo Gutiérrez-de-Terán[1,2*]

[1]Department of Cell and Molecular Biology, BMC, Box 596 and [2]Science for Life laboratory, Uppsala University, SE-751 24, Uppsala, Sweden

[3]Department of Pharmaceutical Chemistry, BMC, Uppsala University, SE-751 23, Uppsala, Sweden

1 Supplementary Figures and Tables

Table S1- RMSD of the ligands along the MD trajectory of the protein-ligand complex used for LIE calculations. Ligands with K_i > 100 µM (i.e. non-binders) are highlighted in italics and denoted with a star. It can be appreciated the higher RMSD values, indicating instability of the binding mode proposed which resulted in lost interactions with the Zn^{+2} coordination cluster

Compound	RMSD of ligand (in Å) +/- SEM
IRAP-wildtype	
6	2.23 ± 0.71
7	1.99 ± 0.63
8	2.46 ± 0.78
9	2.46 ± 0.82
I - IRAP	
6	1.74 ± 0.55
7	2.10 ± 0.66
8	1.50 ± 0.47
9	1.70 ± 0.54
V - IRAP	
6	2.51 ± 0.79
7	2.35 ± 0.74
8	2.62 ± 0.83
9	2.29 ± 0.72
HFI-Series	
15a	*4.68 ± 1.48*
15b	*3.90 ± 1.23*
15c	4.69 ± 1.48

15d[*]	*3.75 ± 1.19*
15e[*]	*4.36 ± 1.38*
15f[*]	*3.90 ± 1.23*
15g[*]	*4.40 ± 1.39*
16a[*]	*3.98 ± 1.26*
16b[*]	*4.25 ± 1.34*
16c	2.15 ± 0.68
16d	2.40 ± 0.76
16e	2.61 ± 0.83
16f	1.97 ± 0.62
16g	1.91 ± 0.60
16h	2.60 ± 0.82
16i[*]	*3.50 ± 1.11*
16j[*]	*3.38 ± 1.07*
16k[*]	*3.46 ± 1.09*
16l	2.65 ± 0.84
16m	2.32 ± 0.73
16n	2.59 ± 0.82
16o	2.26 ± 0.71
16p[*]	*5.45 ± 1.72*
16q	2.33 ± 0.74
16r	1.91 ± 0.60
17a	2.44 ± 0.77
17b	2.04 ± 0.65
17c	1.84 ± 0.58
17d	2.61 ± 0.83
17e	1.91 ± 0.60
17g	1.94 ± 0.61
18d	2.32 ± 0.73
18f	2.01 ± 0.64
18g	2.02 ± 0.64
18h[*]	*5.29 ± 1.67*
